# Do the New Rural Pension Scheme promote the health status of chronic patients in old age? —Evidence from CHARLS 2018 in China

**DOI:** 10.1186/s12889-023-17430-9

**Published:** 2023-12-14

**Authors:** Qihong Liang, Yuxuan Chen, Zheng Zhang, Shengli An

**Affiliations:** Department of Biostatistics, School of Public Health (Guangdong Provincial Key Laboratory of Tropical Disease Research, Guangdong Provincial Key Laboratory of Construction and Detection in Tissue Engineering), Southern Medical University, No.1838 North Guangzhou Avenue, Guangzhou, 510515 People’s Republic of China

**Keywords:** Chronic diseases in the elderly, NRPS, Bayesian networks, Regression discontinuity design, Causal inference

## Abstract

**Background:**

Many researchers have examined the impact of social insurance on health in elderly. However, in most cases, they have only demonstrated correlational results and have not been able to determine causal effects, possibly because confounding biases have not been fully addressed. In this study, we investigated the health effects of the New Rural Pension Scheme (NRPS) on the elderly (age≥60 years old) with chronic diseases in rural areas, and to explore the causal relationship and effects of NRPS and health status.

**Methods:**

This paper used data from the 2018 China Health and Retirement Longitudinal Study (CHARLS) and applied Bayesian networks and fuzzy regression discontinuity design to conduct causal analysis. Bayesian networks were used to explore the causal directed acyclic graphs of factors related to NRPS and health status. Based on the results of Bayesian network, a fuzzy regression discontinuity design was employed to estimate the causal effect of NRPS on health status.

**Results:**

Among rural elderly with chronic diseases, Bayesian network mapping of causal relationships among NRPS, health status and covariates showed that age was a common cause of NRPS receipt and satisfaction with health. The results of the fuzzy regression discontinuity analysis showed that the effect of receiving NRPS on the health status was positive, but there was no statistically significant difference concerning the interval estimates. The results of the subgroup analysis with chronic obstructive pulmonary disease (COPD) and asthma indicated that the effect of NRPS receipt on the health status of elderly people with COPD was positive. There was a statistically significant effect of receiving NRPS on self-rated health description (${\beta }_{1}=3.653,P=0.032$) and health satisfaction (${\beta }_{1}=4.204,P=0.033$) in COPD population and a statistically significant effect of receiving NRPS on health satisfaction in asthma population (${\beta }_{1}=9.844,P=0.008$).

**Conclusion:**

This paper has confirmed the contribution and positive causal effect of NRPS on health status in a subgroup of older adults with COPD and asthma, using the CHARLS database as evidence. Thus, Chinese government should increase the take-up rate of the NRPS to enhance their positive impact on health status of elderly people with chronic diseases in rural areas.

**Supplementary Information:**

The online version contains supplementary material available at 10.1186/s12889-023-17430-9.

## Introduction

Internationally, the proportion of patients suffering from chronic diseases especially the proportion of elderly is gradually increasing with age. In 2015, *the World Report on Ageing and Health* published by stated that chronic diseases in the elderly have become a global epidemic [1]. A large-scale systematic review of chronic diseases and multimorbidity for the elderly over the last 20 years in European and American countries reveals that over half of the elderly suffer from multiple chronic diseases, and the prevalence is increasing sharply among the elderly at advanced ages [2]. Similar results were obtained in a 2016 cross-sectional study applied to elderly population that included Europe, Asia and Africa. Russia had the highest prevalence of multiple chronic diseases (71.9%), whereas China (45.1%) and Ghana (48.3%) had the lowest [3]. Moreover, more than two multimorbidity patterns were found in Spain including “cardio-respiratory” (angina, asthma, and chronic obstructive pulmonary disease), “metabolic” (diabetes, obesity, and hypertension), and “mental-articular” (arthritis and depression) [4]. Similarly in China, over half of Chinese aged 70 or over suffer from multiple chronic diseases in several cross-sectional studies of chronic diseases in older adults [5–7]. Especially in rural areas, the overall health status of rural elderly is not optimistic, with 90.5% of rural elderly suffering from at least one chronic disease [8].

As a high-risk group for chronic diseases, elderly people are more likely to experience chronic disease-related problems that affect their health status, quality of life, health care burden, and mortality. Many studies indicate that chronic disease is one of the risk factors for the deterioration of health status of the elderly. The FINE study (Finland, Italy, Netherlands, Elderly in rural) shows that having more than chronic diseases significantly increased the 10-year mortality risk in all cohorts [9]. Another analysis of the trajectory of chronic diseases reveals that chronic diseases not only affect the current physical function of older adults, but also predict later declines in physical function [10]. A longitudinal study of community-dwelling older adults in China employed a linear mixed effects model to demonstrate that chronic disease is associated with lower health-related quality of life of the elderly [11]. The results in China are consistent with the results of the Gallup World Poll, a survey in more than 160 countries, that having a chronic disease reduces the well-being of older adults [12]. In short**,** chronic health problems have become as dominant a health care burden as infectious diseases, and almost all chronic conditions are strongly related to aging. The burden of elderly with chronic diseases on countries, families and individuals is substantial, both in terms of health and economic burden.

As a means to prevent and reduce chronic disease burdens, the World Health Organization (WHO) published Active Ageing: A Policy Framework in 2002 [13]. And the World Health Day Theme of 2012 was *Adding life to years*. It stressed the importance of taking action on multiple fronts, including strengthening health care service security, since wealth influences utilization of health care services [14]. Brazil, for instance, provided free primary chronic disease medications to older adults attending public health clinics [15]. Similarly, China has also established corresponding social security programs: the New Rural Cooperative Medical Scheme (NRCMS) and the New Rural Pension Scheme (NRPS), the two pillars of its social security system. In 2009, the NRPS was launched throughout all rural areas in China. People over 60-year-old with household registration in rural areas are entitled to a monthly pension. This scheme is aimed at protecting the basic livelihood of elderly in rural residents and thus improving their live [16].

Currently, studies in many countries, especially in high-income countries, have proved that mortality from chronic diseases can be reduced by corresponding measures [17]. A research using data from the Global Burden of Diseases, Injuries, and Risk Factors Study 2019 demonstrates that public health and intervention programs can reduce chronic disease to enhance healthy life expectancy among older adults [18]. In China, it has been proven by a number of studies that NRCMS was not associated with any health outcome of rural elderly [19]. Regarding the impact of NRPS, previous studies only correlated NRPS with the improvement of elderly health outcomes using regression or association analysis [20]. However, there have been very few studies conclusively proving a causal relationship between NRPS and the health of elderly. Thus, it is uncertain whether the implementation of NRPS is an effective mean of improving the health of elderly and prolonging their lives, especially for chronically ill elderly with poor health status. To address this issue, we performed a retrospective observational study of elderly with chronic diseases across China to determine the effect of NRPS receipt on the health status.

In recent years, researches in health care have increasingly focused on causal relationships between variables. Methods of causal inference, such as directed acyclic graphical model and quasi-natural-experiments, are commonly used in medical research. Bayesian Networks (BNs) is based on a directed acyclic graph (DAG), which was first proposed by Pearl Judea in 1987 and has been used for causal exploration, prediction, and. disease diagnosis [21]. The structure learning algorithm of Bayesian Networks mainly includes the constraint-based and the score-and-search-based approach [22]. Tabu search algorithm, which is one of the score-and-search-based approach, it was proposed by Glover in 1986 [23, 24] and has the characteristics of few parameters, simple structure and strong global optimization capability. Meanwhile, the regression discontinuity design (RDD) permits strong causal inference with relatively weak assumptions, which is used to study the outcomes related to an abrupt change when it is impossible to randomize subjects to the conditions before and after the change. An RDD aims to minimize the effect of confounding on the estimated effect of a policy or treatment change [25]. Unobserved confounding is particularly concerning in nonrandomized studies because this bias cannot be completely removed using conventional statistical methods, such as regression or propensity score–based analysis [26]. An RDD attempts to minimize the risk for confounding bias when generating the association between an exposure and the intervention in the outcome of interest [27].

The purpose of this paper is to examine the health impact of NRPS implementation on rural elderly with chronic diseases through BNs and RDD in light of research background and current policies. The rest of the paper is structured as follows. Firstly, a descriptive analysis described basic statistics and main variables of CHARLS. Secondly, a DAG containing various variates was plotted through BNs to explore the causal relationship between health status and NRPS receipt. Thirdly, an RDD was conducted to investigate how NRPS affects health and estimate the magnitude of the causal effect of NRPS receipt on health status. Lastly, different parameter estimation methods in discontinuity regression models and subgroups of the population with chronic disease were compared in order to examine the robustness of the results. Unlike previous correlational studies, this study confirmed the causal relationship and effect of NRPS on rural elderly with chronic diseases. Its objective is to provide some evidence to help promote implementation of the New Rural Pension Scheme and strengthen health management for rural elderly people.

## Method

### Study design and population

This study was a retrospective, observational study from the China Health and Retirement Longitudinal Study (CHARLS), a large interdisciplinary and longitudinal survey project hosted by the National Development Institute of Peking University. The project aims to collect a set of high-quality microdata representative of middle-aged and elderly people (age≥45 years old) in China. CHARLS conducted a national baseline survey in 2011, using probability sampling in proportion to the population size at the country-village-household-individual level to ensure an unbiased and representative sample. By the time of completion of the national follow-up in 2018, the survey had covered a total of 19,000 respondents in 12,400 households.

This study used the latest 2018 national follow-up survey from CHARLS. The samples with household registration in rural were included, while those of other registrations were excluded. Subjects aged ≥45 years old with household registration in rural and suffered from any of the following 14 chronic diseases mentioned in the CHARLS questionnaire: hypertension, dyslipidemia, diabetes, tumor, chronic obstructive pulmonary disease (COPD), liver disease, heart disease, stroke, kidney disease, digestive disease, mental illness, Alzheimer's disease, arthritis rheumatism, and asthma were screened in the analysis. For missing values, we excluded samples containing missing values for the outcome variables and all covariates due to the high number of missing values in these samples. Ultimately, there were 8665 middle-aged and elder adults with chronic diseases living in rural households in this final study cohort. Among them, age above 60 years old are elderly. The data can be downloaded on request from the CHARLS website (http://charls.pku.edu.cn/index.htm).

### Variables

#### The dependent variable

An individual's self-evaluation of his or her health combined with self-rated health score is generally considered an appropriate and effective index for representing the real health status of an individual in many previous studies [28, 29]. Therefore, the self-rated health, which is an is an essential item in the CHARLS questionnaire containing five response options (“Extremely poor”, “Very poor”, “Average”, “Very good”, “Extremely good”) [30], was used to measure subjective health in this study. The assessment score of each response ranges from 1 to 5.

In order to be more reliable, this research adopted satisfaction with health in the CHARLS questionnaire to evaluate the health status of the rural elderly as well. The Gallup World Poll, an large and worldwide survey in more than 160 countries, indicated that psychological wellbeing especially healthy satisfaction was closely linked to health at older ages [31]. In the questionnaire, satisfaction with health was obtained by the answer “How satisfied are you with your health?” with the options including “Not at all satisfied”, “Not very satisfied”, “Somewhat satisfied”, “Very satisfied”, “Completely satisfied”. The degrees of satisfaction with health are assigned with 1–5 points, respectively.

#### The independent variables

The key explanatory variable in this research was whether the subscriber received NRPS. Subjects were divided into two groups based on their answers to the question "Do you currently receive pension benefits from the New Rural Resident Pension?”. In this study, we also adjusted for covariates through extracting subjects’ parameters including age, gender, ethnicity, marriage, residence, education level, number of children, whether to take care of children, number of properties, annual income, smoking status, drinking status and chronic diseases.

### Statistical analysis

#### Univariable analysis

The analysis on the association of the independent variables above with receipt of NRPS was performed for all eligible elderly. Continuous variables were performed as mean and standard deviation (SD) or median and interquartile range (IQR) dependent on whether the normal distribution assumption was satisfied. The Shapiro-wilk test was performed to test the normality of the variables. Categorical variables were performed as numbers and percentages (%). The chi-square tests, Fisher’s exact probability test, independent t-test, and Wilcoxon test were used to compare the difference between group with NRPS receipt and group without NRPS receipt appropriately.

#### Bayesian Network

We used the Bayesian Network (BN) model to explore the causal directed acyclic graphs of factors related to NRPS and health status of the elderly with chronic diseases in rural areas. The BN model was constructed by tabu algorithm, and the probability of each node was estimated using maximum likelihood method based on 15 variables (shown in Table 1). According to the policy, only rural residents who have reached the age of 60 can receive the NRPS. Therefore, we set up a whitelist of paths in our algorithm from age to whether to receive NRPS (age→NRPS). Since the BN model is applicable to discrete random variables, we converted the continuous type variables (age, ethnicity and annual income) used in the study to binomial data types. The conversion rules were as follows: 1) age: Value=1 ( $\ge 60$ years), Value=0 (age < 60 years); 2) ethnicity: Value=1 (Han), Value=0 (Other); 3) annual income: Value=1 ($\ge 960$ RMB), Value=0 (< 960 RMB), because the median annual income in the sample was 960 RMB.

**Table 1:** Basic characteristics between two groups

**Variable**	**Characteristics**	**Total** **(*N=*8665)**	**NRRP=0** **(*N=*5547)**	**NRRP=1** **(*N=*3118)**	***P* Value**
**Health status**					
Self-rated health	Median [IQR]	2.0 [2.0, 3.0]	2.0 [2.0, 3.0]	2.0 [2.0, 3.0]	0.439
Satisfaction With Health	Median [IQR]	2.0 [2.0, 3.0]	2.0 [2.0, 3.0]	3.0 [2.0, 3.0]	0.423
**Running variable**					
Age (year)	Median [IQR]	66.0 [59.0, 73.0]	60.0 [54.0, 69.0]	70.0 [66.0, 74.0]	<0.001
**Economic Status**					
Number of properties	Median [IQR]	2.0 [1.0, 3.0]	2.0 [1.0, 3.0]	3.0 [1.0, 3.0]	0.962
Annual income (RMB, yuan), n (%)	Low income (≤720)	3501 (40.4)	2952 (53.2)	549 (17.6)	<0.001
	Lower middle income (720~1100)	1729 (20.0)	513 (9.2)	1216 (39.0)	
	Middle income (8508.5~12530.2)	1704 (19.7)	703 (12.7)	1001 (32.1)	
	High income (>18051.5)	1731 (20.0)	1379 (24.9)	352 (11.3)	
**Demographic characteristics**					
Gender, n (%)	Male	3025 (34.9)	1899 (34.2)	1126 (36.1)	0.082
	Female	5640 (65.1)	3648 (65.8)	1992 (63.9)	
Ethnicity, n (%)	Han	7954 (91.8)	5034 (90.8)	2920 (93.6)	<0.001
	Others	711 (8.2)	513 (9.2)	198 (6.4)	
Marriage, n (%)	Married	6848 (79.0)	4510 (81.3)	2338 (75.0)	<0.001
	Divorced	451 (5.2)	369 (6.7)	82 (2.6)	
	Widowed	1314 (15.2)	629 (11.3)	685 (22.0)	
	Unmarried	52 (0.6)	39 (0.7)	13 (0.4)	
Residence, n (%)	Town	990 (11.4)	712 (12.8)	278 (8.9)	<0.001
	Rural	7675 (88.6)	4835 (87.2)	2840 (91.1)	
Education level, n (%)	Illiterate	2666 (30.8)	1408 (25.4)	1258 (40.3)	<0.001
	Did not finish primary school	2132 (24.6)	1322 (23.8)	810 (26.0)	
	Home school	16 (0.2)	8 (0.1)	8 (0.3)	
	Elementary school	1944 (22.4)	1323 (23.9)	621 (19.9)	
	Middle school	1476 (17.0)	1154 (20.8)	322 (10.3)	
	High school	381 (4.4)	298 (5.4)	83 (2.7)	
	Vocational school	36 (0.4)	25 (0.5)	11 (0.4)	
	Two-/Three- Year college degree	12 (0.1)	9 (0.2)	3 (0.1)	
	Bachelor’s degree	2 (0.0)	0 (0.0)	2 (0.1)	
Number of children, n (%)	0	66 (0.8)	48 (0.9)	18 (0.6)	<0.001
	1	793 (9.2)	676 (12.2)	117 (3.8)	
	≥2	7806 (90.0)	4823 (86.9)	2983 (95.6)	
Taking care of children, n (%)	Yes	3710 (42.8)	2502 (45.1)	1208 (38.7)	<0.001
**Habit**					
Smoking status, n (%)	Yes	2860 (33.0)	1757 (31.7)	1103 (35.4)	<0.001
Drinking status, n (%)	Yes	1357 (15.7)	823 (14.8)	534 (17.1)	<0.001
**Chronic disease**	Yes				
Hypertension, n (%)	Yes	4052 (46.8)	2397 (43.2)	1655 (53.1)	<0.001
Dyslipidemia, n (%)	Yes	2208 (25.5)	1422 (25.6)	786 (25.2)	0.680
Diabetes, n (%)	Yes	1350 (15.6)	830 (15.0)	520 (16.7)	0.037
Tumor, n (%)	Yes	243 (2.8)	174 (3.1)	69 (2.2)	0.015
COPD, n (%)	Yes	1574 (18.2)	896 (16.2)	678 (21.7)	<0.001
Liver disease, n (%)	Yes	708 (8.2)	474 (8.5)	234 (7.5)	0.098
Heart disease, n (%)	Yes	2119 (24.5)	1242 (22.4)	877 (28.1)	<0.001
Stroke, n (%)	Yes	738 (8.5)	384 (6.9)	354 (11.4)	<0.001
Kidney disease, n (%)	Yes	1072 (12.4)	681 (12.3)	391 (12.5)	0.747
Digestive disease, n (%)	Yes	3591 (41.4)	2338 (42.1)	1253 (40.2)	0.079
Mental illness, n (%)	Yes	236 (2.7)	143 (2.6)	93 (3.0)	0.297
Alzheimer's disease, n (%)	Yes	359 (4.1)	194 (3.5)	165 (5.3)	<0.001
Arthritis rheumatism, n (%)	Yes	4637 (53.5)	2845 (51.3)	1792 (57.5)	<0.001
Asthma, n (%)	Yes	668 (7.7)	359 (6.5)	309 (9.9)	<0.001

#### Fuzzy regression discontinuity design

The impact of NRPS on health cannot be estimated by multiple linear regression owing to its age-based nature. In China, only those aged 60 or older are eligible for NRPS, leading to a surge in NRPS reception rate, with those of advanced age more likely to have received NRPS, while relatively younger elder have held off, which creates a selection bias. As endogeneity may arise due to the presence of selection bias [32], the causal effects of the NRPS on health cannot be adequately analyzed by comparing health situations before and after receipt of the pension. To deal with the endogeneity problem, we adopted a regression discontinuity design (RDD) to analyze causal relationships between receipt of NRPS and healthy status in elderly. RDD, a quasi-random testing method, was first developed by Campell as a way of estimating treatment effects in a nonexperimental setting where treatment is determined by whether an observed “assignment” variable (also referred to in the literature as the “running” variable) exceeds a known cutoff point [33, 34].

In this study, the legal age for NRPS reception was used as the running variable to detect the casual effect of the NRPS on health. Under current policy system in China, NRPS receipt were made on an annual/quarterly basis, and some elderly people whose birth month exceeded the application period for NRPS were not able to receive it on time. In general, the NRPS reception rate increased dramatically at age 60, but it didn't jump from zero to one. Therefore, the analysis in this study was based on a fuzzy regression discontinuity design.

Assumed that Y was the outcome variable indicating the health status, *R* was the running variable with a cutoff point *c*, which represented age in this study, and *T*_*i*_ was the grouping variable of subscriber i. Correspondingly, whether to receive NRPS was considered as the grouping variable in this study. At the cutoff point of the running variable, the probability of an individual being assigned to the treatment group jumped from a to b (0<*a*<*b*<1), thus the probability $0<{\text{Pr}}\left({T}_{i}=1|{R}_{i}>c\right)-{\text{Pr}}\left({T}_{i}=1|{R}_{i}<c\right)<1$ holded [35]. Local nonparametric estimation is commonly used to estimate the causal effect of grouping variables on outcome variables, known as the local average treatment effects (LACT) [36]. The goal was to construct models for the grouping of variables and outcomes with parameters $\alpha =\left({\alpha }_{0},{\alpha }_{1}\right),\beta =({\beta }_{0},{\beta }_{1})$ to be estimated, respectively:

$${T}_{i}={\alpha }_{0}+{\alpha }_{1}{D}_{i}+F\left(S\right)+{\epsilon }_{i}$$

$${Y}_{i}={\beta }_{0}+{\beta }_{1}{T}_{i}+G\left(S\right)+{\varepsilon }_{i}$$

Here, ${D}_{i}$ was an indicator variable on the cutoff point, which followed that $${D}_{i}=\left\{\begin{array}{c}0,{S}_{i}\le 0\\ 1,{S}_{i}>0\end{array}\right.$$ with ${S}_{i}={R}_{i}-c$. F(S), G(S) were polynomials about S and ${\epsilon }_{i}, {\varepsilon }_{i}$ were random error in these models. 

Thus, the LACT can be estimated as the ratio of the change in the elderly's health and the spike in the probability that the elderly was 60 years old through two-stage least squares estimation [37, 38]:

$${\text{LATE}}=\frac{\underset{\varepsilon \to {0}^{+}}{{\text{lim}}}E\left[Y|R=c+\varepsilon \right]-\underset{\varepsilon \to {0}^{-}}{{\text{lim}}}E\left[Y|R=c+\varepsilon \right]}{\underset{\varepsilon \to {0}^{+}}{{\text{lim}}}E\left[D|R=c+\varepsilon \right]-\underset{\varepsilon \to {0}^{-}}{{\text{lim}}}D\left[D|R=c+\varepsilon \right]}.$$

Moreover, mean square error (MSE) was used for the estimation and selection of the optimal bandwidth in above models. To facilitate coefficient interpretation, a quadratic function was used for local curve fitting while triangular method was used for the Kernel weighting method. A two-sided *P* value <0.05 was an indicator of statistical significance.

#### Sensitive analysis and additional analysis

To ensure the robustness of the results, we employed different bandwidths including MSERD, MSESUM, MSETWO for parameter estimation as sensitive analysis. Meanwhile, a subgroup analysis of 14 chronic diseases mentioned previously was conducted. Moreover, an ordinal stepwise Logistic regression was further conducted to examine the association between the time interval and amount of NRPS receipt and outcomes after considering the covariates.

#### Software

All analyses were performed by R software v4.2.1 (R Foundation for Statistical Computing, Boston, MA, USA). The BN model was run in bnlearn package. The BN graph and BN inference model were drawn by Netica (5.18). The optimal bandwidth selection was achieved through the rdbwselect function in the rdrobust package.

## Results

### Descriptive statistics

There were 8665 individuals who lived in rural areas met inclusion criteria during the study period, 3025 (34.9%) were men and 5640 (65.1%) were women. Elderly characteristics and the univariate analysis of NRPS receipt were summarized in Table 1. Only 3118 (36.0%) of the study population received NRPS. The mean age was 66.0 years (IQR: 59.0~73.0), and the histogram of the age distribution was shown in Figure S1 (Supplementary Fig. S1). There were 7675 (88.6%) elderly living in rural areas. Only 2.7% of people suffer from mental illness, with arthritis rheumatism being the most common (53.5%), followed by hypertension (46.8%).

### Bayesian Network

**Fig. 1:**
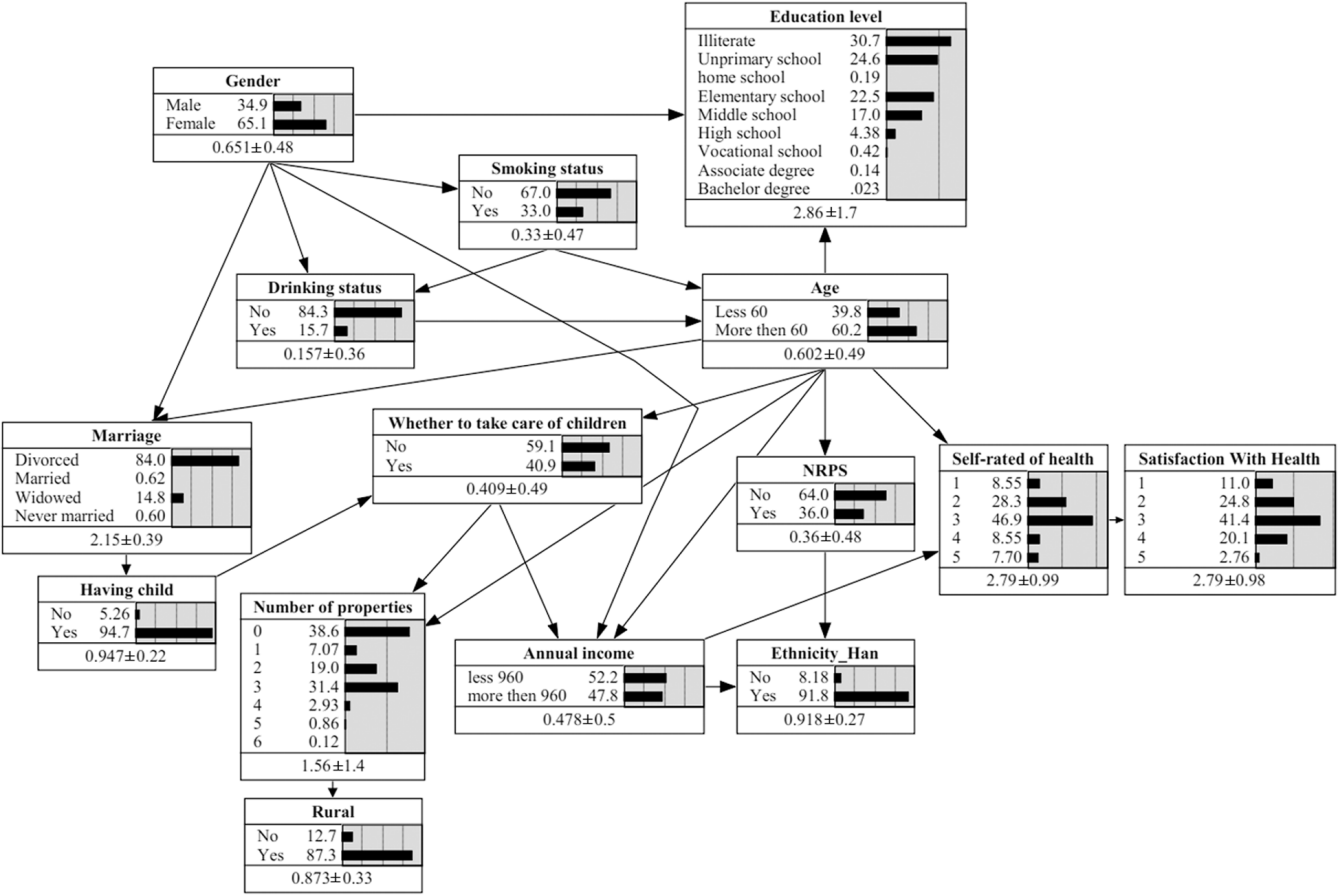
Bayesian network constructed by tabu algorithm and its prior probability

Figure 1 shows that the BN model contains 15 nodes and 24 arcs. Each node represented one variable, and the arc between connected nodes indicated the cause-effect relations among the nodes. The number in the node indicated the marginal probability of each node. For example, the marginal probability of being not at all satisfied with self-health (self-rated health=1) was ${P}_{({\text{self}}-\mathrm{rated health}=1)}=0.0855$.

Age, annual income and satisfaction with health were directly connected to self-rated health. Age and annual income were the parents of self-rated health. Supplementary Table S1 represents the Conditional Probability Table (CPT) for self-rated health, from which we can see that the probability of being not at all satisfied with self-health (self-rated health=1) is highest when age >= 60 and annual income < 960 RMB, with the probability of 10.74%. Satisfaction with health was a child node of self-rated health, that is, the level of self-rated health is related to health satisfaction. The remaining twelve variables were indirectly related to self-rated health.

In this study, age served as the parent node for self-rated health and whether to receive NRPS, that is, age affected whether to receive NRPS and self-rated health, and NRPS could also be inferred to be associated with self-rated health, although a direct causal relationship did not exist. Figure S2 indicates the sensitivity analyses. At the receiving NRPS with the probability of 100%, the probability of being not at all satisfied with self-health (self-rated health=1) increased slightly from 8.55% to 9.31%, which may affect by age. For this reason, we considered age as a running variable in the regression discontinuity design in order to determine whether to receive NRPS had a true impact on self-rated health.

### Fuzzy regression discontinuity design

#### The discontinuity at the cutoff point and internal validity test of running variable

**Fig. 2:**
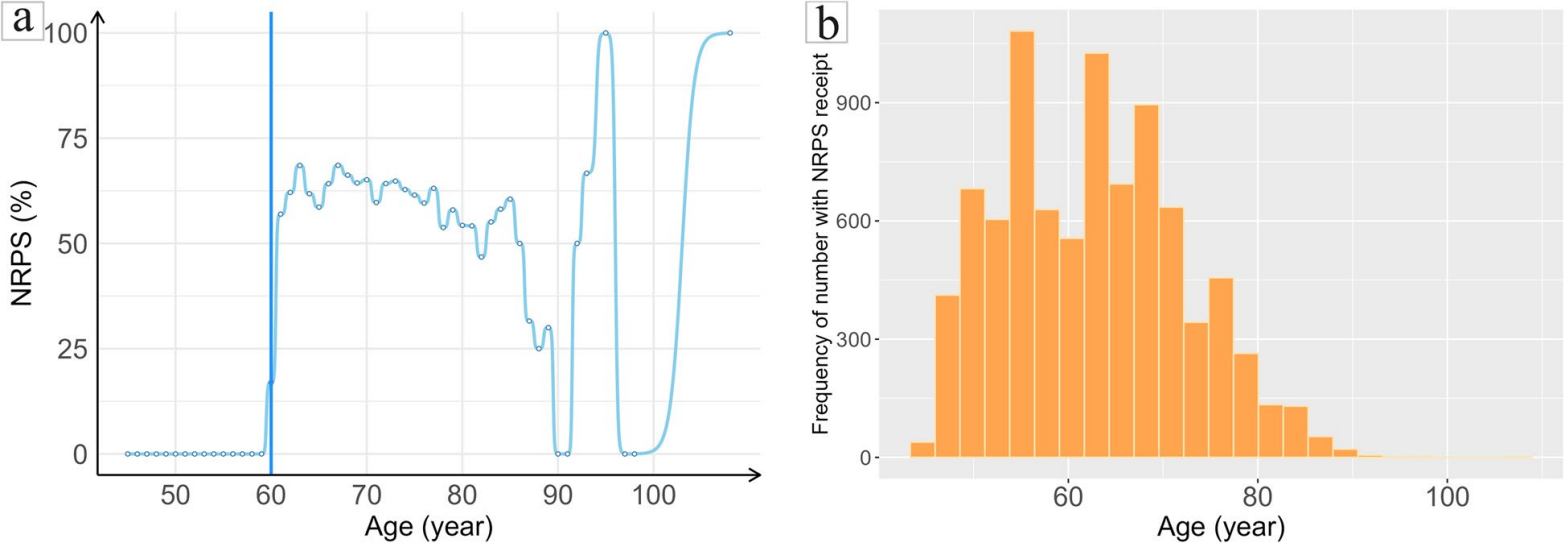
The change and frequency of the New Rural Pension Scheme (NRPS) receipt at different ages. **a** The change of the New Rural Pension Scheme (NRPS) receipt at different ages. **b** The frequency of the New Rural Pension Scheme (NRPS) receipt at different ages

A valid fuzzy regression discontinuity design relies on two main assumptions: the assumption of discontinuity and the assumption of internal validity [27, 39]. The first assumption requires a discontinuity in the probability of grouping at the cutoff point, which was verified by depicting the change of the NPRP receipt rate at different ages. As shown in Fig. 2a (left), there was a notable jump in the NPRP receipt rate before and after the age 60, but not with the probability from 0 to 1. The second assumption of internal validity requires continuity in potential outcomes as a function of the grouping variable around the cutoff point [38], indicating that in the absence of NRPS, the running variable should not change at the cutoff point. Though this assumption cannot be tested directly, a histogram of age was able to show the distribution of age among subjects in this study sample. Figure 2b (right) illustrates that the age distribution at the cutoff point (60-year-old breakpoint) was uniform and did not jump, demonstrating that age was not affected by individual differences, which satisfied the continuity and meets the internal validity condition.

#### The results of the regression models

**Fig. 3:**
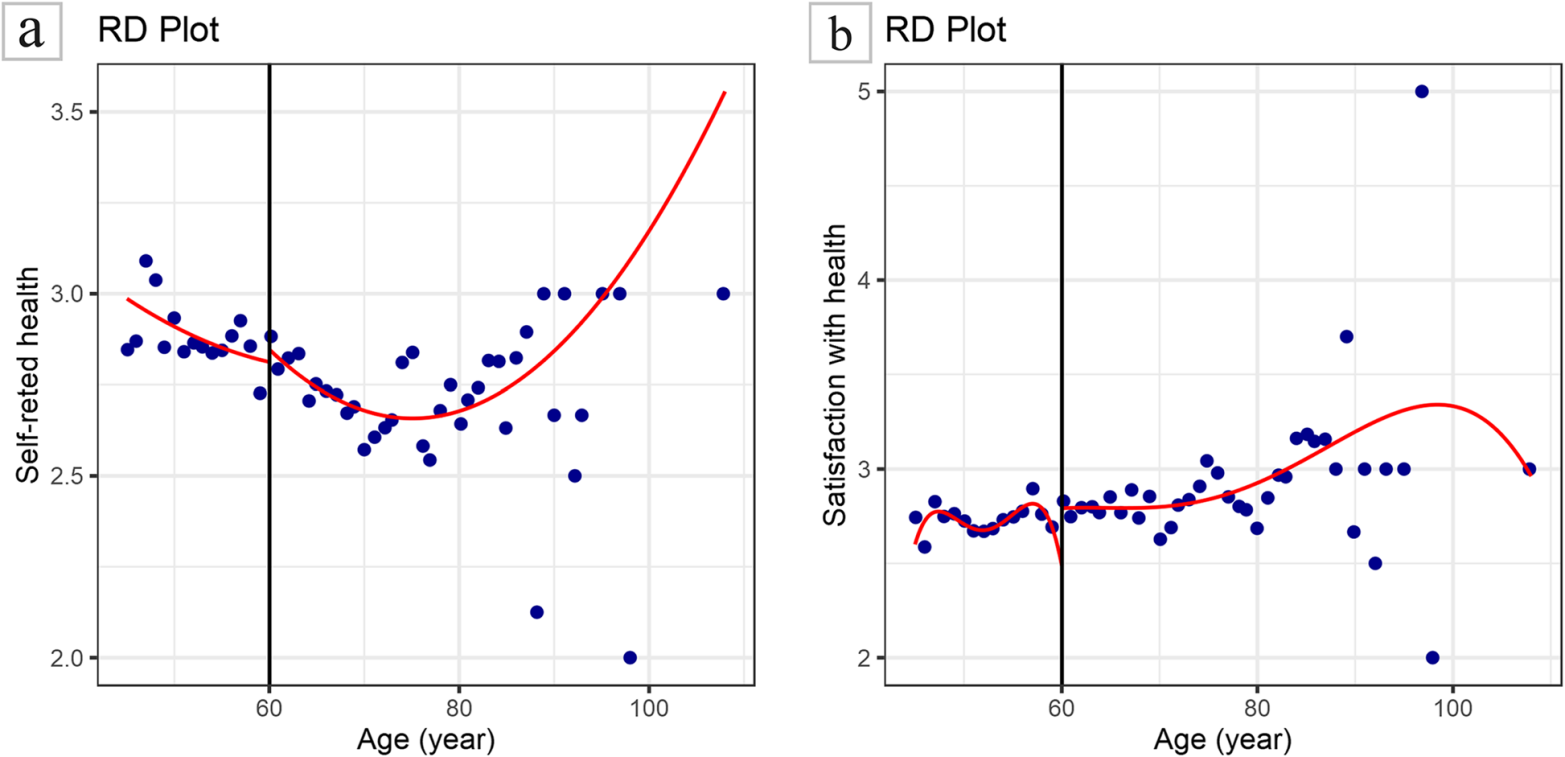
Association between age and health status. **a** Association between age and self-rated of health. **b** Association between age and satisfaction with health

The self-rated health (*Y*_1_) and satisfaction with health (*Y*_2_) were used as outcome variables to fit quadratic models, respectively. Demographic characteristics besides age in Table 1 were included as covariates in the model. The estimated results of applying fuzzy RDD are reported in Table 2. The nonparametric estimation results of models with and without control variables including demographic characteristics, economic status, etc. are reported respectively. The regression results showed that the grouping variable had no significant effect on the self-rated health before and after adjusting covariates (LACT=1.829, *P*=0.089; LACT=1.344, *P*=0.101). A similar result was found in the model with *Y*_2_ (LACT=-4.911, *P*=0.420; LACT=-4.963, *P*=0.329). In contrast, the point estimate of NRPS receipt on self-rated health was more than 1 (1.829, 1.344)), suggesting that NRPS receipt may benefit health status among elderly with certain chronic disease subgroups. Figure 3 depicts the curves after adjusting the covariates.

**Table 2:** Effect of NRPS receipt on health status

Outcome	Model^a^	Bandwidth	Parameter	Coefficient	Standard Error	*Z*	*P*	95% *CI*
*Y*_1_	Unadjust	MSESUM	Conventional	1.839	1.080	1.702	0.089	[-0.278, 3.956]
			Robust	-	-	1.776	0.076	[-0.232, 4.723]
	Adjust	MSESUM	Conventional	1.344	0.820	1.640	0.101	[-0.262, 2.951]
			Robust	-	-	1.717	0.086	[-0.243, 3.683]
*Y*_2_	Unadjust	MSESUM	Conventional	-4.911	6.087	-0.807	0.420	[-16.841, 7.019]
			Robust	-	-	-0.828	0.408	[-18.183, 7.382]
	Adjust	MSESUM	Conventional	-4.963	5.085	-0.976	0.329	[-14.930, 5.004]
			Robust	-	-	-0.973	0.331	[-15.974, 5.378]

^a^Unadjusted: Inclusion of grouping variables (whether to receive NRPS) only; Adjusted: Adjusted for covariates including gender, ethnicity, marriage, residence, education level, number of children, whether to take care of children, number of properties, annual income, smoking status, drinking status and chronic diseases

#### Robustness test

As part of sensitive analysis, we used different bandwidths including MSERD, MSESUM, and MSETWO to conduct a re-estimation of the regression models. Table 3 demonstrates the coefficients estimates under different bandwidths. There was no significant impact of the NRPS receipt both on the self-rated of health (LACT=0.825,* P*=0.124; LACT=1.344, *P*=0.101) or the self-rated of health (LACT=-3.379, *P*=0.349; LACT=-4.963, *P*=0.329) before and after adjustment. This sensitive analysis indicated that the methods for parameter estimation were sound and the results were robust.

**Table 3:** Effect of NRPS receipt on health status with different bandwidths

Outcome	Bandwidth	Parameter	Coefficient	Standard Error	*Z*	*P*	95% *CI*
*Y*_1_	MSETWO	Conventional	0.825	0.537	1.538	0.124	[-0.226, 1.877]
		Robust	-	-	1.659	0.097	[-0.192, 2.309]
	MSERD	Conventional	1.344	0.820	1.640	0.101	[-0.262, 2.951]
		Robust	-	-	1.717	0.086	[-0.243, 3.683]
*Y*_2_	MSETWO	Conventional	-3.379	3.605	-0.937	0.349	[-10.444, 3.686]
		Robust	-	-	-0.981	0.327	[-11.370, 3.786]
	MSERD	Conventional	-4.963	5.085	-0.976	0.329	[-14.928, 5.003]
		Robust	-	-	-0.973	0.331	[-15.983, 5.377]

#### Subgroup analysis in chronic diseases

According to the above results, the receipt of NRPS had no significant impact on health status in the chronic disease population, possibly due to the fact that the effect of NRPS receipt in different diseases was masked by the overall effect in the chronic disease population. For that reason, 14 common chronic diseases were subjected to subgroup analyses. Furthermore, there was a significant effect of NRPS on health status in COPD and asthma cohorts. Therefore, further subgroup analysis was performed in COPD with 1579 individuals and asthma cohorts with 668 individuals, respectively.

**Fig. 4:**
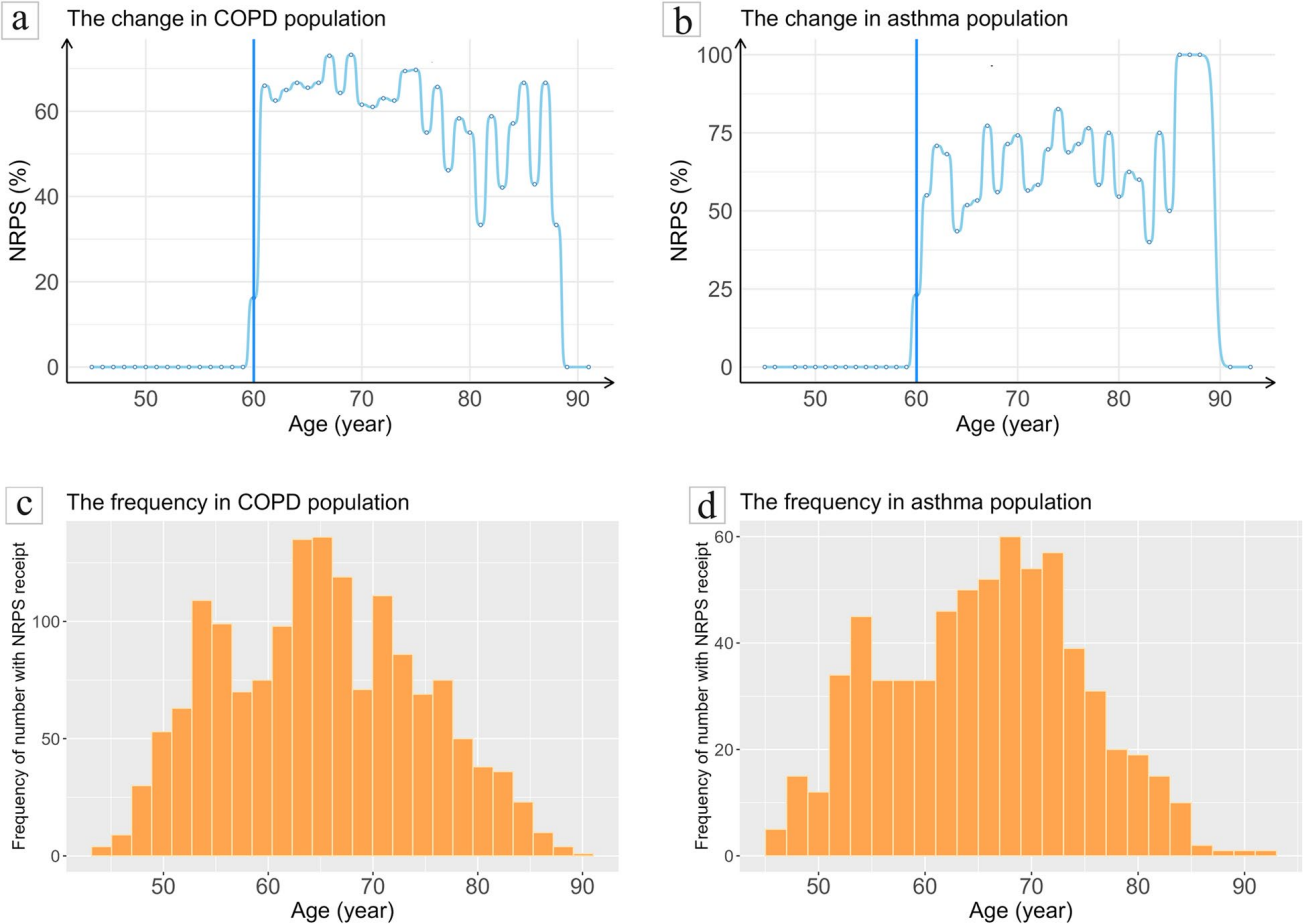
The change and frequency of the New Rural Pension Scheme (NRPS) receipt at different ages. **a** The change of the New Rural Pension Scheme (NRPS) receipt at different ages in COPD population. **b** The change of the New Rural Pension Scheme (NRPS) receipt at different ages in asthma population. **c** The frequency of the New Rural Pension Scheme (NRPS) receipt at different ages in COPD population. **d** The frequency of the New Rural Pension Scheme (NRPS) receipt at different ages in asthma population

Similarly, the assumptions about the discontinuity at the cutoff point and internal validity test of running variable were verified by plotting change curves and histograms. The receipt rates of NRPS at different ages were plotted separately in COPD and asthma subgroups. As depicted in Fig. 4a and b, a jump with probability clearly not from 0 to 1 at age 60 occurs in both subgroups, signifying the presence of a cutoff point at age 60. Histograms in Fig. 4c and d portrays the distribution of age in elderly with COPD and asthma. The age distributions in two subgroups at around 60 years old were even and without jumps, meeting the internal validity condition.

**Fig. 5:**
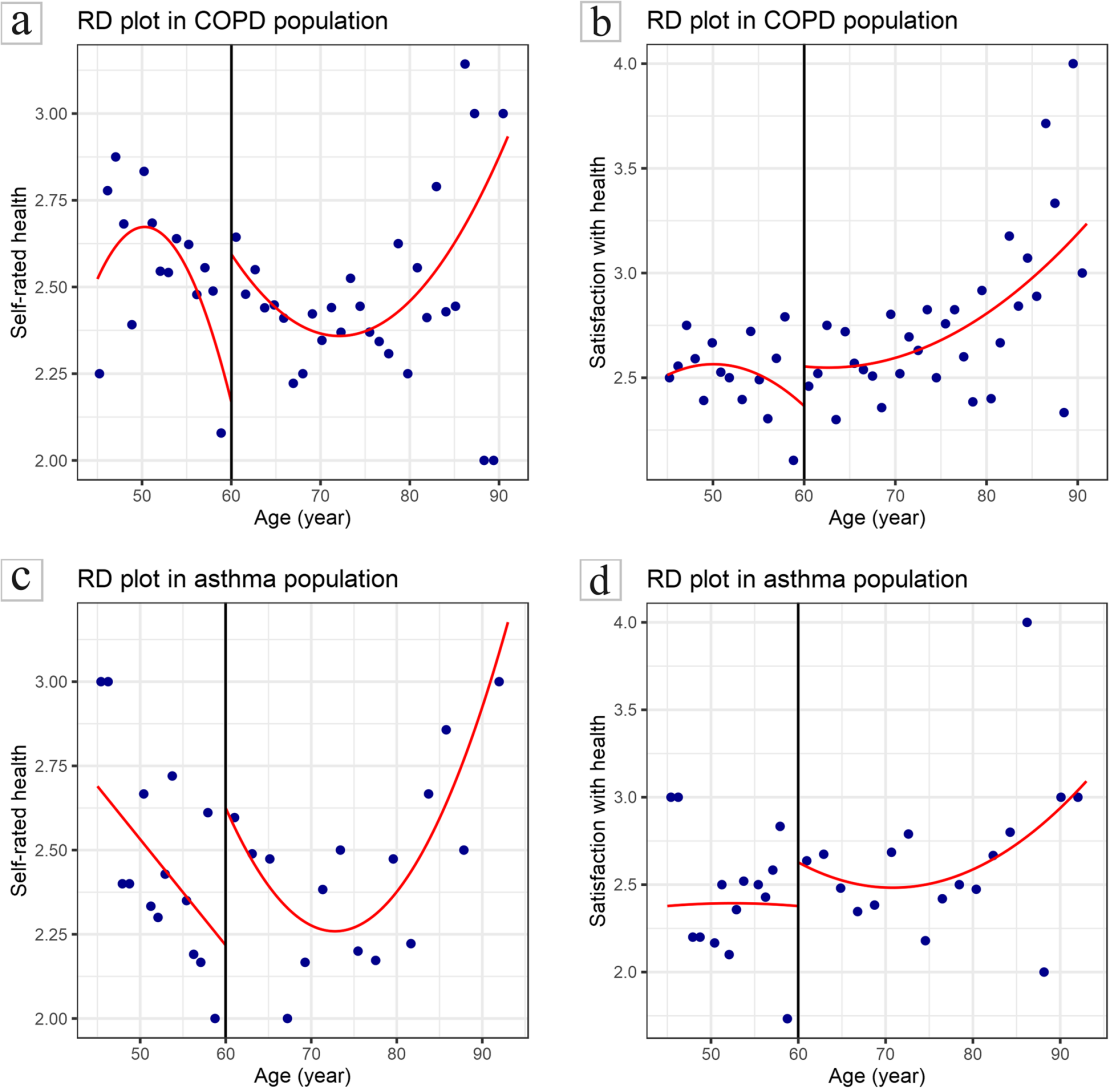
Association between age and health status in two subgroups. **a** Association between age and self-rated of health in COPD population. **b** Association between age and satisfaction with health in COPD population. **c** Association between age and self-rated of health in asthma population. **d** Association between age and satisfaction with health in asthma population

Table 4 reports the estimated results. Among the elderly with COPD, there was significantly difference in the effect of whether to receive NRPS on self-rated of health and satisfaction with health before adjusting for covariates (LACT=3.691, *P*=0.037; LACT=4.638, *P*=0.025), and the receipt of NRPS significantly improved the health status including self-rated of health (LACT=3.653, *P*=0.032) and satisfaction with health (LACT=4.204, *P*=0.025) after adjustment. Based on these results, receiving NRPS had a positive effect on health status among elderly people with COPD. Likewise, among the elderly with asthma, the effect of NRPS receipt on self-rated of health was not significant whether adjusting for covariates or not. On the contrary, the effect on satisfaction with health was significantly positive in both models. Moreover, the LACT of the covariate adjustment on satisfaction with health was 9.844 (*P*=0.008), higher than the LACT of 7.568 (*P*=0.005) before adjustment, demonstrating that receiving NRPS significantly improved the satisfaction with health of elderly asthmatics after excluding the covariate effect. In conclusion, NRPS receipt was beneficial to satisfaction with health in elderly with asthma. In addition, Fig. 5 portrays the fitting curves of health status after adjusting the covariates in two subgroups.

**Table 4:** Effect of NRPS receipt on health status in two subgroups

Subgroup	Outcome	Model^a^	Bandwidth	Parameter	Coefficient	Standard Error	*Z*	*P*	95% *CI*
COPD	*Y*_1_	Unadjust	MSETWO	Conventional	3.691	1.769	2.086	0.037	[0.224, 7.157]
				Robust	-	-	2.224	0.026	[0.523, 8.292]
		Adjust	MSETWO	Conventional	3.653	1.701	2.148	0.032	[0.319, 6.986]
				Robust	-	-	2.323	0.020	[0.694, 8.189]
	*Y*_2_	Unadjust	MSERD	Conventional	4.638	2.071	2.240	0.025	[0.579, 8.697]
				Robust	-	-	2.611	0.009	[1.583, 11.113]
		Adjust	MSERD	Conventional	4.204	1.977	2.126	0.033	[0.329, 8.079]
				Robust	-	-	2.365	0.018	[0.971, 10.382]
Asthma	*Y*_1_	Unadjust	MSETWO	Conventional	4.064	2.423	1.677	0.093	[-0.685, 8.812]
				Robust	-	-	1.869	0.062	[-0.263, 11.026]
		Adjust	MSESUM	Conventional	3.743	2.056	1.821	0.069	[-0.287, 7.773]
				Robust	-	-	1.992	0.046	[0.077, 9.577]
	*Y*_2_	Unadjust	MSETWO	Conventional	7.568	2.686	2.818	0.005	[2.304, 12.831]
				Robust	-	-	2.951	0.003	[3.276, 16.237]
		Adjust	MSETWO	Conventional	9.844	3.682	2.673	0.008	[2.627, 17.061]
				Robust	-	-	2.966	0.003	[4.163, 20.393]

^a^Unadjusted: Inclusion of grouping variables (whether to receive NRPS) only; Adjusted: Adjusted for covariates including gender, ethnicity, marriage, residence, education level, number of children, whether to take care of children, number of properties, annual income, smoking status, drinking status and chronic diseases

A sensitive analysis, a re-estimation of the regression models with different bandwidths as reported in Table 5, is conducted in two subgroups as well. In the COPD subgroup, the effects under different bandwidths were similar to the above results, suggesting the robustness of the result that NRPS had a positive effect on health. However, in the asthma subgroup, self-rated of health and satisfaction with health were not significantly affected by receiving NRPS with different bandwidths. Even though the effect at different bandwidth choices was not significant, the point estimates of the LACT on both outcomes were significantly higher than 0, indicating a positive trend. According to some literature, the bandwidth is affected by sample size and should be selected based on the minimum bandwidth when the sample size is insufficient, otherwise large bias will result [40, 41]. Consequently, the small sample size of the asthma subgroup may be contributing to the instability of sensitivity analysis, which had also been occurred in similar studies [28]. In this study, as bandwidth selection was based on optimal criteria, the results of the regression models remained valid.

**Table 5:** Effect of NRPS receipt on health status in two subgroups with different bandwidths

Subgroup	Outcome	Bandwidth	Parameter	Coefficient	Standard Error	*Z*	*P*	95% *CI*
COPD	*Y*_1_	MSERD	Conventional	4.898	2.312	2.119	0.034	[0.367, 9.429]
			Robust	-	-	2.127	0.033	[0.449, 10.959]
		MSESUM	Conventional	4.940	2.357	2.096	0.036	[0.320, 9.559]
			Robust	-	-	2.104	0.035	[0.397, 11.167]
	*Y*_2_	MSETWO	Conventional	2.233	0.735	3.040	0.002	[0.793, 3.674]
			Robust	-	-	3.157	0.002	[1.020, 4.360]
		MSESUM	Conventional	3.664	1.675	2.187	0.029	[0.381, 6.948]
			Robust	-	-	2.343	0.019	[0.722, 8.119]
Asthma	*Y*_1_	MSERD	Conventional	4.218	2.863	1.473	0.141	[-1.394, 9.829]
			Robust	-	-	1.669	0.095	[-0.973, 12.129]
		MSESUM	Conventional	4.890	3.410	1.434	0.152	[-1.794, 11.574]
			Robust	-	-	1.630	0.103	[-1.282, 13.967]
	*Y*_2_	MSERD	Conventional	24.514	26.657	0.920	0.358	[-27.731, 76.760]
			Robust	-	-	1.181	0.238	[-22.254, 89.679]
		MSESUM	Conventional	23.722	25.562	0.928	0.353	[-26.378, 73.822]
			Robust	-	-	1.164	0.244	[-21.625, 84.874]

#### Additional analysis considering the time interval and amount of NRPS receipt

The stepwise ordinal Logistic regressions removed the time interval and amount from the optimal model for self-rated health and satisfaction with health (Supplementary Table S2). According to the results, the time interval and amount of NRPS receipt may not have a significant impact on the relationship between NRPS and health. For self-rated health, the score was reduced by alcohol consumption compared to non-drinkers. The self-rated health of subjects with high annual incomes was better than that of subjects with low annual incomes. For satisfaction with health, female, smokers, alcoholics, and middle annual income earners rated satisfaction with health lower, while high annual income earners rated satisfaction with health higher.

## Discussion

The research cohort for this paper consisted of 8665 rural elderlies with chronic diseases from CHARLS 2018. In this study, Bayesian networks and fuzzy regression discontinuity design were used to conduct an empirical study of the effects of NRPS on the health status of the elderly with chronic diseases. It was indicated that receiving NRPS had a positive effect on health status, but the effect was relatively modest without statistically significant, and it was only statistically significant in some specific subgroups of patients. The results were generally consistent with several previous similar studies in China, which could not fully control for statistical bias despite using complex models for their analyses [20, 42]. To summarize, this research differed from previous studies in three significant ways. Firstly, only rural elderly with chronic diseases were eligible in the study cohort, whereas previous studies hadn't distinguished between urban and rural elderly, who receive varying types of pension scheme [43]. Some studies had included rural older adults, with subgroup analyses of region or income level, yet had failed to consider chronic disease as a significant subgrouping factor [16]. Secondly, the pension of interest in this study was NRPS, as most previous studies had forced on the relationship between health and medical pension like the New Rural Cooperative Medical Scheme (NRCMS). Among elderly, the results of the same database CHARLS indicated that NRCMS positively impacted the health status and satisfaction [44]. Similar results were found in other databases including the Chinese Longitudinal Healthy Longevity Survey [45]. Considering that medical pension can be utilized directly for medical treatment, these were clearly common-sense results. Thirdly, this study estimated the causal effects of NRPS on health by combining Bayesian networks with RDD. Contrary to this, previous studies have employed regression models like Logistic regression [42], dynamic fixed-effects regression [46] or conducted a propensity score matching [47], RDD alone for effect estimation. Most of these studies could infer only a relationship between NRPS and health [28].

Using Bayesian network analysis, we identified age as a common cause of whether to receive NRPS and self-rated health, although there was no direct causal relationship between the two variables. This is consistent with Cheng’s study [20], in which a fixed-effect model with instrumental variable correction was used to estimate the causal effect of NRPS income on self-rated health of the elderly. Possible explanations are that the effect of NRPS on health status will probably be linked or masked by age.

This paper further estimated the causal effect of NRPS on health status using fuzzy discontinuity regression. It found a positive effect of receiving NRPS on health status in chronically ill older adults, but the difference between the interval estimates was not statistically significant. This may be due to the complexity of the type of chronic disease, resulting in an insignificant overall effect value. This contrasts with Tang’s findings [42]. In Tang’s study, the logit model and stepwise regression method were used to analyze and compare the influence of economic support on self-rated health, where socioeconomic support (part of economic support) includes a variety of different endowment insurance, while in this study, only the effect of NRPS on the rural chronically ill elderly was considered. Moreover, Tang's study detected an association between economic support and self-rated health, not a causal link.

We further checked whether the effects were heterogeneous between different chronic diseases. Ultimately, NRPS had a positive impact on the health status of older adults with COPD and asthma.

Among older adults with COPD, receiving NRPS improved both self-rated health description and health satisfaction. Similar relationships for older adults with asthma. Those receiving NRPS reported better health satisfaction. One possible explanation is that NRPS could improve the health of older adults with COPD and asthma by reducing the financial burden on families. NRPS is primarily designed to provide income for the elderly to reduce their financial burden as part of a social security system [48]. Rural elderly people can receive financial assistance through the NRPS for chronic disease treatment, so that their health can be improved, especially for rural elderly who have long-term chronic diseases and are difficult to cure. There is a reason for this, as older adults living in rural areas face long treatment courses and high treatment costs for chronic diseases [47]. In addition, it has cost an average Chinese household 33–40% of its income to treat COPD, ranging from US$1964 to $3449 per patient per year [49]. Similar findings have been found in asthma.

Additionally, we examined the health effects of NRPS on older adults with other chronic diseases such as hypertension, diabetes, and heart disease using fuzzy regression discontinuity design. However, the results were not significant. This may be due to the fact that several chronic diseases are covered by the New Rural Cooperative Medical System (NRCMS), although reimbursement rates vary among them. NRCMS reimburses 80% of patients' essential drug expenditures, such as those for hypertension or diabetes [50]. Therefore, NRCMS could greatly reduce the financial burden on rural elderly with hypertension and diabetes. NRCMS reimburses low rates for drugs used to treat COPD and asthma because it still focuses its reimbursement coverage on inpatient expenses. For the elderly with these two chronic diseases, the NRPS could alleviate the medical and financial pressure of the patients to a greater extent, so the direct causal effect of the NRPS was more significant in the COPD and asthma populations.

Our results showed that the NRPS participation rate among rural seniors with chronic diseases was only 33.6%, much lower than the participation rate of the entire population in CHARLS 2018 [48]. Considering the positive effects of NRPS on chronically ill rural elderly, government should work to improve pension benefits to achieve better health outcomes for rural older adults. For instance, elderly people with chronic diseases should be subsidized for their premiums to increase the take-up rate of pension schemes, thereby reducing the financial burden and improving the health status of the chronically ill elderly.

## Innovation

The advantage of this paper is that the Bayesian network and fuzzy regression discontinuity design can be used as evidence for causal inference, and the conclusions drawn are of a higher level of argument than those of regression models and correlation analysis. In this paper, we use Bayesian networks to provide exploratory hypotheses and fuzzy regression discontinuity design to estimate causal effect values, and these two causal inference methods are cascaded.

Another advantage of this paper is that this study considered elderly with chronic diseases who were always in poorer health and at high risk of mortality. By using subgroup analyses to examine the impact of the NRPS on the health status of different chronic disease populations, the conclusions drawn are more pertinent.

## Limitations

There are several limitations to our study. First of all, the health status of people is difficult to measure accurately. Both the self-rated health score and the satisfaction with health score were considered as indicators of health status, but this measurement may still be subject to subjective bias. Secondly, we used data from the CHARLS 2018 survey to describe the latest effects of NRPS on the health status of the elderly with chronic diseases. Therefore, we cannot provide information with longitudinal data. Thirdly, it should be pointed out that due to the limited data availability, the paper does not include information on the health status of older adults with chronic diseases in rural areas after 2020, i.e., during the epidemic of COVID-19. These questions further need to be answered by offering more theoretical and empirical evidence. For example, follow-up studies can be conducted based on the latest CHARLS survey data and consider longitudinal changes in the data over time to provide more comprehensive evidence on the implementation of the NRPS policy in the context of the new coronavirus.

## Conclusion

This study tries to investigate the impact of NRPS on health status for elderly people with chronic disease in rural China. The univariate analyses showed that self-rated health and satisfaction with health did not significantly differ between the groups of recipients and non-recipients of NRPS before adjusting for any covariates using RDD. Compared to those who did not receive the NPRS, among those who did receive the NPRS, there were fewer low-income people, more Han Chinese, fewer married people, more people living in rural areas, lower levels of education and fewer children. And more elderly who smoked, drank and suffered from chronic diseases including (hypertension, COPD, heart disease, stroke, Alzheimer's disease, Arthritis rheumatism) received NRPS. The empirical results have shown that NRPS has a positive causal effect on the health status of older adults with COPD and asthma. Further, the stepwise ordinal Logistic regressions indicated that annual income has a positive effect on health status. The causal path from annual income to health status was demonstrated in a Bayesian network as well. Based on the results of this study, the Chinese government should increase the take-up rate of the New Rural Pension Scheme, so that it can improve the health status of the elderly with chronic diseases in rural areas, especially those suffering from COPD and asthma.

### Supplementary Information

**Additional file 1:** **Table S1.** Conditional Probability Table (CPT) for self-rated health. **Table S2.** Additional analysis of stepwise ordinal Logistic regressions for health status. **Fig. S1.** Age distribution of 8665 subjects. **Fig. S2.** Sensitivity analysis of Bayesian network on the New Rural Pension Scheme (NRPS) receipt.

## Data Availability

Data is publicly available. See: https://charls.charlsdata.com/pages/Data/2018-charls-wave4/zh-cn.html.

## Abbreviations

BNs: Bayesian Networks
CHARLS: China Health and Retirement Longitudinal Study
CHP: China Pulmonary Health
COPD: chronic obstructive pulmonary disease
DAG: directed acyclic graph
LACT: local average treatment effects
MSE: mean square error
MSERD: MSE-optimal bandwidth selector for the regression discontinuity treatment effect estimator
MSESUM: MSE-optimal bandwidth selector for the sum of regression estimates
MSETWO: two different MSE-optimal bandwidth selectors (below and above the cutoff) for the regression discontinuity treatment effect estimator
NRPS: the New Rural Pension Scheme
NRCMS: the New Rural Cooperative Medical Scheme
RDD: regression discontinuity design
WHO: the World Health Organization
