# Supplementary figures and images for: Do the New Rural Pension Scheme promote the health status of chronic patients in old age? —Evidence from CHARLS 2018 in China
Source: BMC Public Health. 2023 Dec 14;23:2506. doi: 10.1186/s12889-023-17430-9 (PMC10720147; doi:10.1186/s12889-023-17430-9)

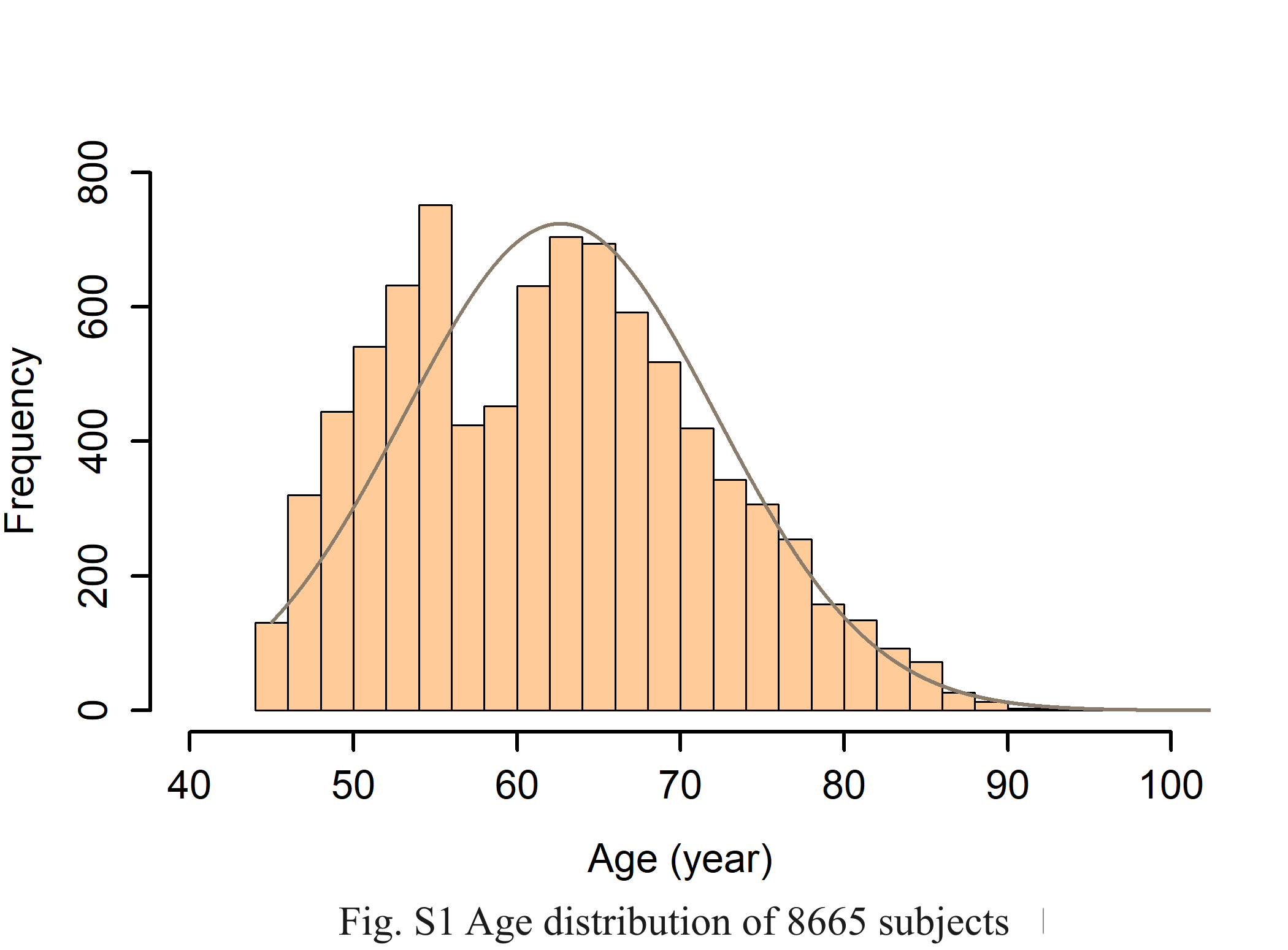

Supplement: Supplementary file 1 — Additional file 1: Table S1. Conditional Probability Table (CPT) for self-rated health. Table S2. Additional analysis of stepwise ordinal Logistic regressions for health status. Fig. S1. Age distribution of 8665 subjects. Fig. S2. Sensitivity analysis of Bayesian network on the New Rural Pension Scheme (NRPS) receipt. [file 12889_2023_17430_MOESM1_ESM.zip › Supplementary material/FigureS1.tiff]

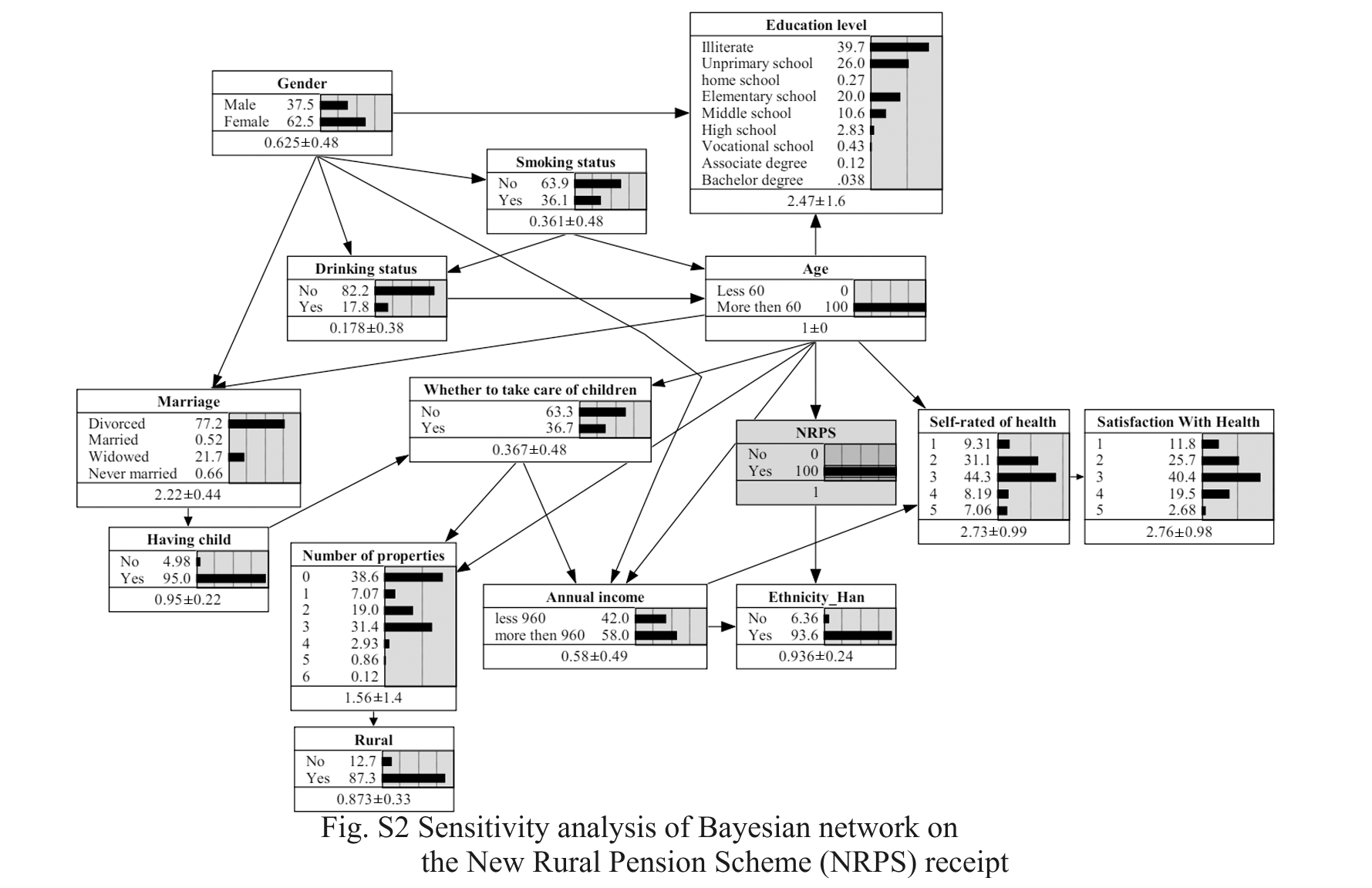

Supplement: Supplementary file 1 — Additional file 1: Table S1. Conditional Probability Table (CPT) for self-rated health. Table S2. Additional analysis of stepwise ordinal Logistic regressions for health status. Fig. S1. Age distribution of 8665 subjects. Fig. S2. Sensitivity analysis of Bayesian network on the New Rural Pension Scheme (NRPS) receipt. [file 12889_2023_17430_MOESM1_ESM.zip › Supplementary material/FigureS2.tif]
